# Training With an Evaluation Framework: Outcomes From a Biosecurity Training Intervention in Commercial Poultry Farms in Tamil Nadu, South India

**DOI:** 10.1002/vms3.70385

**Published:** 2025-05-05

**Authors:** Alagarsamy Alagesan, Ganesh Janarthanan, Arumugam Balakrishnan, Paramasivam Vigneshwaran, Vasudevan Gowthaman, Papaiyan Kumaravel, Fiona Tomley, Ayona Silva Fletcher, Thippichettipalayam Ramasamy Gopala Krishna Murthy, Samuel Masilamoni Ronald

**Affiliations:** ^1^ Poultry Disease Diagnosis and Surveillance Laboratory Veterinary College and Research Institute Campus Namakkal Tamil Nadu India; ^2^ Department of Veterinary Microbiology Madras Veterinary College Chennai Tamil Nadu India; ^3^ Veterinary College and Research Institute Udumalpet Tamil Nadu India; ^4^ Department of Pathobiology and Population Sciences Royal Veterinary College London UK; ^5^ Department of Clinical Sciences and Services Royal Veterinary College London UK

**Keywords:** adoption behaviour, bio‐security practices, knowledge gain, poultry farmers

## Abstract

The study aimed to investigate the assessment of farm profiles, knowledge gain, and adoption behaviours of biosecurity practices following a training intervention in commercial poultry farms in Tamil Nadu. A total of 89 farmers from commercial desi (32), layer (30), and broiler (27) farms participated in the training program. The biosecurity assessment used an evaluation framework with a two‐day training program. Participants completed pre‐ and post‐training surveys to measure knowledge gained during the training. Additionally, we conducted a follow‐up evaluation of adoption behaviours after 90 days of training intervention. Questionnaire data were analysed using paired sample *t*‐test, chi‐square, and regression analysis. Results revealed that 89% of the trainees were male, 90% were in the age group between 21 and 60 years, and 88% had a secondary education level or higher. Further, 56.2% of farmers had 5–20 years of experience in poultry farming, and 46.1% of farmers revealed that the significant source of income is from poultry farming and agricultural practices. A pre‐ and post‐survey data comparison showed that all the farmers had significant knowledge gain (*p* < 0.01) in all the categories of structural and operational biosecurity practices immediately after the training. The farmer's educational qualification significantly influences the knowledge gain except for dead bird disposal (*p* < 0.05). Commercial desi and layer farmers have more pre‐existing knowledge compared to broiler farmers. The broiler farmers showed the highest knowledge gain compared to layer and commercial desi farmers. Still, there was no significant difference between knowledge gain among different types of poultry farmers (*p* > 0.05). The adoption behaviour measured after 90 days significantly increased in all categories (*p* < 0.05) except for rodent and pest control. This comprehensive study provided valuable insights regarding farmers’ existing knowledge and the impact of training on some behavioural changes to improve biosecurity. The study concluded that a tailored training program is essential to educate small‐scale producers about biosecurity measures to prevent poultry food‐borne diseases.

## Graphical abstract

This study examined the profiles of 89 poultry farmers in Tamil Nadu, focusing on their knowledge gain and the adoption of biosecurity practices through training programme. A significant improvement in knowledge was observed (*p* < 0.01), mainly among broiler farmers, who demonstrated the considerable knowledge gains. While there was a significant increase in the adoption of biosecurity measures across all the areas, except pest control. It is understood that customised training is essential for effective enhancement of biosecurity in poultry farming.

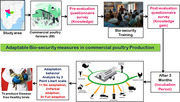

## 1 Introduction

The poultry industry has become the world's leading source of efficient, high‐quality animal protein providers. Poultry and poultry products are essential and cost‐effective protein sources compared to other animal‐based food sources (Conan et al. 2012). Poultry meat and eggs stand out favourably among other animal products regarding protein content, amino acid balance, energy, and micronutrients (Aiyedun et al. 2018; Parikh et al. 2022). There are an estimated 851.8 million chickens in India. In 2023, India's broiler meat production reached around 5 million metric tons. This growth is driven by increasing urbanisation, higher disposable incomes, and a shift towards protein‐rich diets.

The Indian poultry market's value is approximately 28.18 billion USD, projected to grow at a CAGR of 8.1% from 2024 to 2032 (DAHD 2022). The Food and Agricultural Organization of the United Nations (FAO) recommends a daily requirement of 65 to 75 g of total protein, with 40% (36 g) coming from animal sources. However, minimal demand for animal protein in India would be set at 20 g per capita per day, compared to the current availability of 10 g (Muthukumar et al. 2021). The Food and Agriculture Organization (FAO) predicts that poultry meat consumption will quadruple from 10 to 18 kg per capita/year by 2030 (FAO, 2006). This underscores the crucial role of the poultry industry in India's nutritional security. The production of chicken meat needs to be increased significantly to secure India's nutritional needs, which can be accomplished through the expansion of poultry farming. The majority of Indian families rely on chicken and poultry products for both food and income security (Scudiero et al. 2023).

The proportions of broiler, layer, and desi chicken farmers vary in Tamil Nadu state based on factors such as market demand, availability of resources, and consumer preferences. Broiler and layer farming are more prevalent due to the high demand for meat and eggs. Desi chicken farming, though historically significant, has a smaller proportion of commercial operations but remains relevant in rural and backyard settings, especially for cultural or traditional reasons. However, proportions can fluctuate over time based on market dynamics and evolving agricultural practices. Commercial desi chickens, also known as ‘desi’ chickens, are a breed that embodies resilience and adaptability. Indigenous to the region and well‐adapted to local conditions, they are popular in smallholder farming. Their hardiness and ability to thrive in various climates make them a promising asset to the poultry industry. Desi chickens often boast good meat quality, disease resistance, and adaptability. In India, farmers crossbreed or selectively breed chickens for enhanced traits like faster growth and higher egg yield while making efforts to preserve their genetic purity.

Poultry diseases and their associated costs are significant obstacles to sustainable chicken production (Byaruhanga et al. 2017). Diseases reduce productivity, resulting in financial losses for farms and companies. These losses manifest as reduced egg production, lower meat quality, increased production costs related to clinical treatments, and higher flock mortality rates. Some poultry diseases are highly infectious or transmissible epidemics that can spread globally, causing severe economic and public health consequences (Lysholm et al. 2022). To address these challenges, farmers must implement strict biosecurity practices on their poultry farms.

Biosecurity is a critical component and management tool in a successful poultry production system. It is not just a set of measures but a necessity for disease prevention in the poultry industry. According to the World Organization of Animal Health, biosecurity is a set of management and physical measures designed to reduce the risk of introducing, establishing, and spreading animal diseases, infections, or infestations to, from, and within an animal population (Moya et al. 2021; Renault et al., 2021). They prevent disease transmission both directly between animals and indirectly between farms (Ellis‐Iversen et al. 2011). Adopting biosecurity (structural and operational) practices helps to reduce the risk of introducing pathogens into a farm (Guinat et al. 2020). It entails designing and implementing actions to protect domestic chicken flocks from introducing harmful organisms (Ismael et al. 2021). Recent outbreaks of zoonotic diseases that are transmitted from poultry to humans, such as highly pathogenic avian influenza (HPAI), Non‐typhoidal Salmonella (NTS), and campylobacter species in various parts of the world, have affected both wild and domestic birds and impacted the economics, health, and welfare (Charostad et al. 2023; Thomson and Seitzinger 2019). At the same time, farmers have some pre‐existing knowledge of internal and external biosecurity practices in poultry management.

Nevertheless, they need to follow strict biosecurity practices in their farms due to their awareness of the implications of food‐borne pathogens. Considering the high prevalence of poultry zoonoses of public health concern, biosecurity measures for disease prevention are needed in small and medium‐scale poultry farms, which contribute significantly to poultry production in rural India. Implementing these measures not only protects the health of the birds but also leads to significant economic benefits for the farmers.

Training evaluation involves measuring the success or failure of a training intervention by assessing changes in learners' knowledge and practices. Various methodological approaches exist for evaluating educational and training interventions. The most widely used method is Kirkpatrick's four‐level evaluation model, initially proposed in 1959 (Kirkpatrick 1983). The model continues to be helpful and applies in various contexts (Alsalamah and Callinan 2022). The model has been used to evaluate training in many environments, including farmer training (Diab 2015) and professional training (Kinnison et al. 2023). The Kirkpatrick model assesses impact at the individual level, encompassing reaction (level 1), learning (level 2), and behaviour change (level 3), as well as the overall impact on the organisation and society (level 4).

The present study aims to investigate poultry farmers'

1. Existing knowledge and practices in poultry production and biosecurity.
2. Evaluate knowledge gained immediately after a training intervention.
3. Assess behavioural changes to biosecurity practices three months after the training intervention program.
4. Evaluate training interventions and outcomes using the Kirkpatrick model.

## 2 Materials and Methods

### 2.1 Study Sites

**FIGURE 1:**
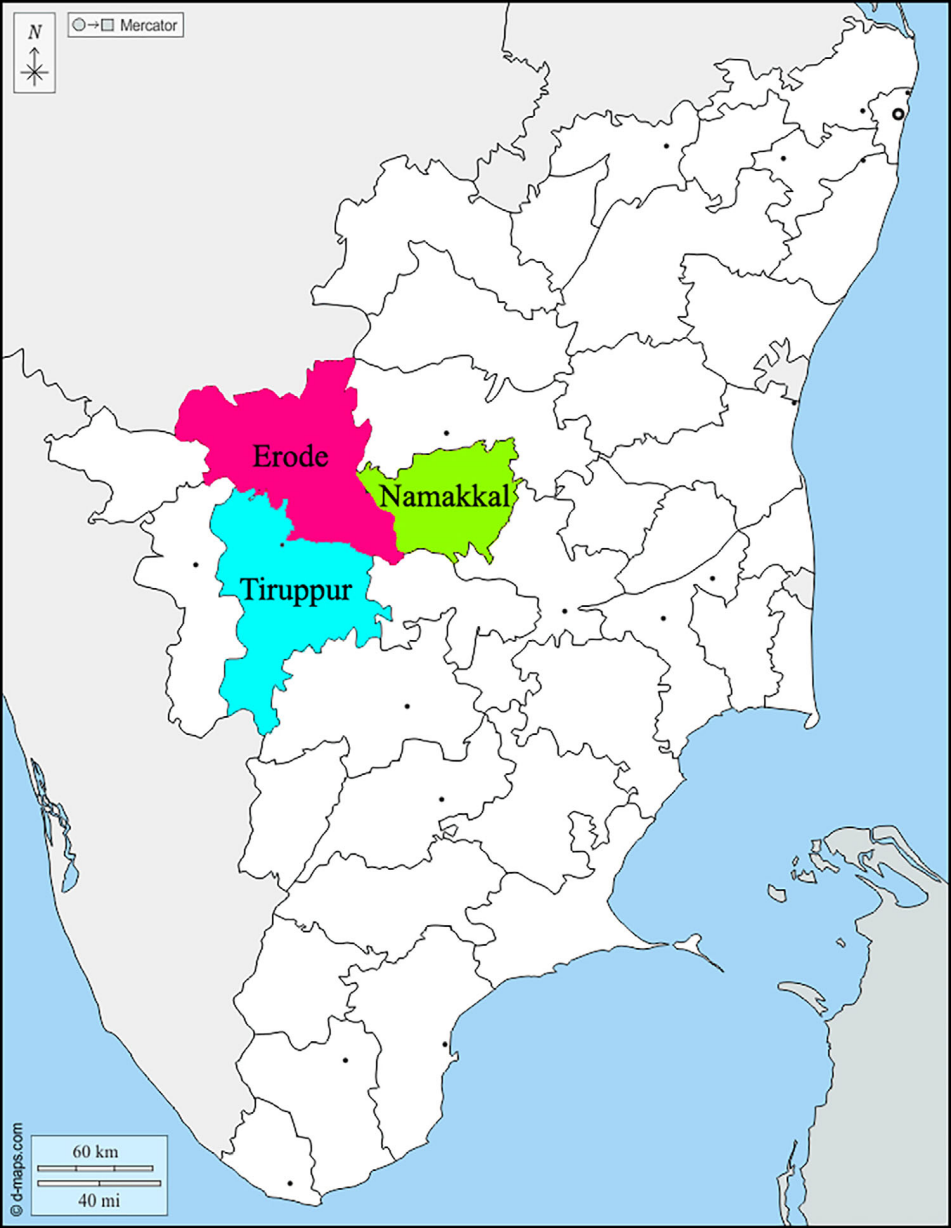
Geographical representation of sampling locations in western agro‐climatic zones of Tamil Nadu, India.

The study was conducted in three major poultry‐producing regions, namely Tiruppur, Namakkal, and Erode districts of Tamil Nadu, South India (Figure 1). Poultry farming plays a vital role in the economy of these regions. The Namakkal region occupies the number one position in layer farming in the country, with a population of more than 70 million commercial layers, producing 50 to 60 million eggs daily. Tiruppur and Erode regions are the leading commercial broiler and commercial desi chicken producers. As per the 20th Livestock Census, 2019, the total poultry population in the country has increased from 729.21 million in 2012 to 851.8 million in 2019, registering an increase of 16.81%. Out of a total poultry population of 851.81 million, 120.80 million chickens are reared in Tamil Nadu, mainly in Namakkal, Erode, and Tiruppur regions. Hence, the training programs organised in these regions targeted specific farmer groups in each region for this study.

### 2.2 Selection of Target Population

The number and size of the farms determined the target populations for the study. This was done to ensure that the farmers were better able to provide the information required to achieve the study objectives. Farmers sometimes did not participate in this training program due to their unavailability. Alternative to this, farm workers who are well‐versed in their operations, such as farm managers and farm supervisors, are assigned by the farm owners to attend this training program. According to the environmental guidelines for poultry farms (2021), the Central Pollution Control Board (CPCB), Government of India, has categorised them as small (5000–25,000 birds), medium (25,000–100,000 birds), and large (more than 100,000 birds) scale poultry producing farmers. The study was carefully designed to include all three categories of representative poultry farmers, farm managers, and farm supervisors from the study areas. The poultry farmers were considered an important data source about the present status of the poultry production enterprise in Tamil Nadu in South India.

### 2.3 Sample Size and Sampling Method

Two survey teams, each consisting of two enumerators, visited each region on the same day and spent four days in each region. Each team visited different subdistricts and poultry farms in production initially identified with the help of a local poultry farmers association or members of the poultry veterinary community. Once teams arrived at the location, enumerators visited different farms individually to select farmers. Snowball sampling was also applied to reach additional respondents by asking farmers or by approaching people who live near the farms, and the study included a total of 93 farmers.

### 2.4 Questionnaire Development

The study was designed as an intervention program with a pre‐test, post‐test, and a 3‐month post‐evaluation of the bio‐security adoption measures. The survey tools were questionnaires and an adoption behaviour scorecard.  The following sections describe the questionnaire and the development of the adoption behaviour score. A comprehensive questionnaire was designed to measure the knowledge gained and practices of the participating farmers. In consultation with subject matter specialists and social scientists, the questionnaire consisted of 30 questions spanning various elements of biosecurity knowledge and practices. This questionnaire was used for pre‐ and post‐evaluation data collection. A pilot study was conducted with twenty farmers (broiler, layer, and commercial desi) in the non‐sample region. The questionnaire was refined and finalised for the study based on the results.

Farmers' socioeconomic profiles and farm‐related information were also collected during the study. The questionnaire was translated into the local dialect of Tamil language and provided to the farmers so that they could adequately comprehend the questions and provide appropriate and reliable responses. The complete training data (pre‐ and post‐evaluation) was collected between June and August 2023. The final questionnaire was categorised into nine subgroups, namely, Farm fencing and visitors' entry (1), Rearing practices (2), Cleaning and disinfection (3), Chick procurement and brooding (4), Poultry diseases and vaccines (5), Antibiotic usage (6), Farm manure disposal (7), Dead bird disposal (8), and Rodent/ Wild bird control (9). These nine subgroups comprised of 23 questions and were filled by the farmers with the help of a project team. A total of 23 questions were chosen to assess their knowledge, attitude, and practices.

### 2.5 Adoption Behaviour Scorecard

The adoption behaviour score was given to each farm, and the result was calculated using the method outlined by Dorea et al. (2010). A grading system was developed to characterise each farm's overall degree of biosecurity practices. The project staff calculated this grading system by observing each farm and comparing it across study strata. The biosecurity score only includes behaviour related to everyday activities. All were assigned scores for frequency of adoption for each measure from the original questionnaire data, preserving the three‐point Likert scale categories (from 1 to 3 for each item corresponding to no adoption (1), partial adoption (2), and full adoption (3)). As summed up, the farms adopting more practices and using them more frequently received higher scores. In the course of the present investigation, 12 practices were used to measure the adoption level, including the nine subgroups measured in the pre‐ and post‐training questionnaire and some additional ones, such as farm fencing, restricted entry, provision of foot and vehicle bath, maintenance of stock, cleaning and disinfection of sheds, use of disinfectants in water, poultry diseases and vaccination, antibiotic usage, farm manure disposal, dead bird disposal, rodent and wild bird control. These biosecurity approaches include both structural and operational components. Structural biosecurity refers to the physical construction, design, and maintenance of a facility to prevent entry of disease vectors and facilitate compliance with operational biosecurity practices. Operational biosecurity involves risk assessments and mitigation of risk through management practices, including implementation of and compliance with standard operating procedures (SOPs) designed to prevent the introduction of the AI virus onto farm premises. However, operational biosecurity was again re‐classified as internal and external biosecurity practices (Elhassan et al. 2022).

### 2.6 Training and Evaluation Program

The training program was planned for three days with a pre‐test, post‐test, and 3‐month post‐evaluation of adoption measures.  After the first training event in Tiruppur district, it was decided that two‐day training programs should be conducted in Namakkal and Erode districts. This was due to the difficulty of retaining farmers three days away from their farms. The training programs were conducted in each region and led by the project team, which included resource persons who are field experts in poultry science, veterinary pathology, and veterinary microbiology from Tamil Nadu Veterinary and Animal Sciences University (TANUVAS).

On day one, the participants completed the pre‐test questionnaire, which measured the existing basic knowledge of poultry management and biosecurity measures. The training methods used brief lectures on biosecurity practices and implementation strategies for intensive poultry production and farm management through narrated PowerPoint presentations, video clippings, handouts/pamphlets/brochures, and detailed pictures in the local language. At the end of the first day, there were discussions with every farmer to share their knowledge in front of field veterinarians, and the individuals were encouraged to ask questions about the training. On day two, the training included on‐farm practical training, and the project team led field visits to poultry farms to discuss biosecurity practices such as farm fencing, foot and vehicle bath, entry and exit maintenance, cleaning and disinfectants, antibiotic usage, rodent and wild bird control, dead bird disposal, and farm manure disposal. Farmers completed the post‐test questionnaire at the end of the third day for the Tiruppur district and the second day for the Namakkal and Erode districts. Farmers who participated in this training program were provided with skill demonstration training kits, which comprised water sanitising tablets, face masks, sprayers, disinfectants, and hand sanitizers to create awareness of implementing biosecurity.

### 2.7 Data and Statistical Analysis

All the collected data from the questionnaires were entered into an Excel spreadsheet, cleaned, and exported for statistical analysis (IBM SPSS Version 23.0 for Windows). Data on demographic and socioeconomic profiles of binary and categorical variables were used to analyse descriptive statistics and chi‐square tests through cross tabulation method. The questionnaire data were checked for normal distribution and analysed using the paired sample *t*‐test to determine the farmers' knowledge gain. In contrast, binary logistic regression was used to assess how farmers' education influences their knowledge gain. For that, primary education and illiterate farmers were considered to have no education and received a score of zero. Those with secondary and higher education were considered educated farmers and received a score of one. Based on the binary variables, binary‐logistic regression was performed. Biosecurity adoption behaviour analyses were carried out using the cross‐tab method. The level of adoption was determined based on farmers' responses, which were then totalled to obtain an individual adoption score.

## 3 Results

### 3.1 Demographic Analyses

**FIGURE 2:**
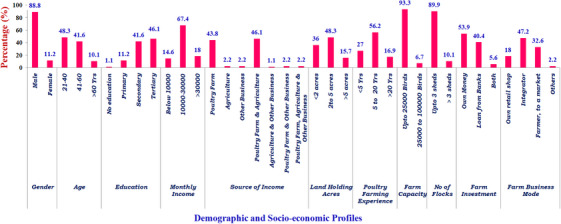
Demographic and socioeconomic profiles of the participants of the training intervention (*n* = 89).

Among the 89 farmers who attended the training program, 32 were from commercial desi farms, 27 were from commercial broiler contracts, and 30 were commercial layer farmers from the respective districts of Tiruppur, Erode, and Namakkal (Table 1). The socioeconomic profiles of the farmers revealed that 88.8% of males and 11.2% of females participated in the training program. Farmers' age groups ranged from 21–40 years (48.3%), 41–60 years (41.6%), and ≥60 years (10.1%). The educational background of the majority of participants was tertiary education (46.1%), followed by secondary education (41.6%), primary education (11.2%), and 1.1% with no formal education. In terms of revenue sources, the combined activities of poultry farming and agriculture were the most significant (46.1%) proportion, followed by poultry farming alone (43.8%) (see Figure 2). Based on the number of birds, three categories can be applied: small <2 acres land, medium 2 to 5 acres, and large >5 acres. Most poultry farmers had medium and small land holdings (84.3%), followed by extensive land holdings (15.7%). Most farmers were concentrated within small and medium land‐holding categories and had small farms (93.3%). Similarly, most respondents (56.2%) had five to twenty years of experience, while 16.9% had over 20 years of experience in the poultry business. About 27% of the participants had less than five years of experience in poultry farming. Regarding farm sizes, most poultry farm operators have (93.3%) managed farms with less than 25,000 birds, while only 6.7% reared 25,000–100,000 birds. Moreover, the farm size varied based on the type of birds reared. Usually, commercial layer farms have more birds compared to commercial broilers and commercial desi.

**TABLE 1:** Data on farmers interviewed and invited for attending the biosecurity training program in three different category of farms in major poultry producing regions of Tamil Nadu.

S. No	Location/region	Farm categories	No. of total farmers interviewed	No. of farmers answered	No of farmers refused	Total no. of participants involved in this training program
1	Namakkal	Layer farms	31	30	1	30
2	Tiruppur	Broiler farms	28	27	1	27
3	Erode	Commercial desi farms	34	32	2	32
Total no. of participants			93	89	4	89

### 3.2 Assessment of Knowledge Gained From the Training Participants

According to the poultry rearing system, poultry farms are categorised as layer, broiler, and commercial desi farms. A total of 23 questions were used to evaluate their Knowledge, each with a value of 3 points, resulting in a maximum possible mean score of 69 points for all 23 questions related to farm management and biosecurity practices in the poultry sector. The analysis of the paired sample *t*‐test revealed that the farmers had obtained significantly more knowledge after the training (Kirkpatrick level 2) (*p* < 0.01). The Knowledge gained score was calculated as follows:

Knowledge gained score (mean ± SE) = (Post‐training score) – (Pre‐training score)

Knowledge gained is categorised as

1. high: between 2 and 3/3
2. moderate: between 1.5 and 2/3
3. low: between 1.5 and 1/3
4. minimal: less than 1/3

#### 3.2.1 Knowledge Gained on Commercial Broiler Farmers

Farmers have good pre‐existing knowledge regarding rearing practices (2.44/3) and distance between sheds (2.56/3). Therefore, these two topics showed minimal knowledge gain at 0.44/3 and 0.44/3, respectively. Compared to this, the highest level of knowledge gained by commercial broiler farmers was in antimicrobial resistance (2.22/3), wild bird menace and control (2.22/3), and farm fencing (2.07/3) (Table 2). Knowledge gained was moderate (between 1.5 and 2 /3) on antibiotics usage (1.93/3), disposal of dead birds (1.89/3), ensuring chick quality (1.85/3), ethno veterinary practices (1.78/3), manure disposal (1.67/3), ensuring feed and water quality (1.67/3), location of poultry farms (1.67/3), disinfectants used in foot and vehicle bath (1.59/3), entry of vehicles (1.56/3), and water sanitisation (1.52/3). Farmers had a low level of knowledge gain (less than 1.5–1/3) in stocking period intervals (1.22), cleaning of sheds (1.37), wastewater disposal (1.19), vaccination (1), antibiotics (1.37), and rodent control (1.22). The knowledge gained was minimal (less than 1/3) in poultry diseases (0.74/3).

**TABLE 2:** Knowledge score (mean ± SE) analysis on broiler farmers regarding biosecurity practices before and after training intervention.

S. No.	Items	Mean score before the training	Mean score after the training	Knowledge gain	*p* value
1	Farm fencing (3)	0.78 ± 0.09	2.85 ± 0.07	2.07 ± 0.07	0.00
2	Restricted entry (3)	1.04 ± 0.08	2.59 ± 0.09	1.56 ± 0.13	0.00
3	Rearing practices (3)	2.44 ± 0.23	2.88 ± 0.11	0.44 ± 0.21	0.04
4	Distance between sheds (3)	2.56 ± 0.21	3.00 ± 0.00	0.44 ± 0.21	0.04
5	Stocking period interval between batches (3)	1.44 ± 0.29	2.66 ± 0.18	1.22 ± 0.29	0.00
6	Location of poultry farms (3)	0.56 ± 0.23	2.22 ± 0.26	1.67 ± 0.37	0.00
7	Disinfectants used in foot and vehicle bath (3)	1.11 ± 0.08	2.70 ± 0.09	1.59 ± 0.13	0.00
8	Cleaning of sheds (3)	1.19 ± 0.08	2.55 ± 0.09	1.37 ± 0.11	0.00
9	Waste water disposal (3)	1.07 ± 0.09	2.26 ± 0.09	1.19 ± 0.14	0.00
10	Ensuring chick quality (3)	0.52 ± 0.19	2.37 ± 0.09	1.85 ± 0.19	0.00
11	Ensuring feed and water quality (3)	1.00 ± 0.12	2.66 ± 0.09	1.67 ± 0.16	0.00
12	Water sanitisation (3)	0.96 ± 0.06	2.48 ± 0.09	1.52 ± 0.13	0.00
13	Clinical signs/diseases (3)	1.59 ± 0.19	2.33 ± 0.15	0.74 ± 0.25	0.01
14	Vaccination (3)	1.52 ± 0.26	2.52 ± 0.09	1.00 ± 0.29	0.00
15	Ethnoveterinary practices (3)	0.78 ± 0.09	2.55 ± 0.09	1.78 ± 0.12	0.00
16	Usage of antibiotics (3)	1.11 ± 0.19	2.52 ± 0.09	1.41 ± 0.20	0.00
17	Mention some antibiotics (3)	0.52 ± 0.16	1.88 ± 0.09	1.37 ± 0.23	0.00
18	Method of usage (3)	0.78 ± 0.08	2.70 ± 0.09	1.93 ± 0.14	0.00
19	AMR (3)	0.00 ± 0.00	2.22 ± 0.12	2.22 ± 0.12	0.00
20	Manure disposal (3)	0.59 ± 0.09	2.26 ± 0.09	1.67 ± 0.12	0.00
21	Dead bird disposal (3)	1.00 ± 0.08	2.89 ± 0.06	1.89 ± 0.11	0.00
22	Rodent control (3)	1.04 ± 0.11	2.26 ± 0.09	1.22 ± 0.11	0.00
23	Wild bird menace & control (3)	0.22 ± 0.09	2.44 ± 0.09	2.22 ± 0.14	0.00
	Overall score (69)/total marks obtained	23.81 ± 1.14	57.85 ± 0.63	34.04 ± 1.36	0.00

#### 3.2.2 Knowledge Gained on Commercial Layer Farmers

Commercial layer farmers gained less knowledge than commercial broiler farmers and did not reach high or moderate levels. The knowledge gained was low on wild bird menace and control (1.47/3), followed by the location of poultry farms (1.40/3), antimicrobial resistance (1.33/3), ensuring feed and water quality (1.30/3), water sanitisation (1.10/3), vaccination (1.10/3), antibiotics (1.07/3), disinfectants used in foot and vehicle bath (1.07/3), ethno veterinary practices (1.0/3), and disposal of dead birds (1.0/3) (Table 3). Knowledge gain was minimal for antibiotic treatment (0.97/3), farm fencing (0.93/3), rodent control (0.90/3), vehicle entry (0.90/3), stocking period interval (0.90/3), manure disposal (0.87/3), wastewater disposal (0.83/3), ensuring chick quality (0.83/3), distance between sheds (0.80/3), antibiotic usage (0.47/3), cleaning of sheds (0.67/3), and poultry diseases (0.73/3). They did not gain any knowledge regarding rearing practices (0/3).

**TABLE 3:** Knowledge score (mean ± SE) analysis on commercial layer farmers.

S. No.	Particulars	Mean score before the training	Mean score after the training	Difference in score	*p* value
1	Farm fencing (3)	1.53 ± 0.09	2.47 ± 0.09	0.93 ± 0.12	0.00
2	Restricted entry (3)	1.8 ± 0.12	2.7 ± 0.09	0.9 ± 0.12	0.00
3	Rearing practices (3)	3 ± 0	3 ± 0	0.00 ± 0.00	0.00
4	Distance between sheds (3)	1.8 ± 0.27	2.6 ± 0.19	0.8 ± 0.25	0.03
5	Stocking period interval between batches (3)	1.8 ± 0.27	2.7 ± 0.17	0.9 ± 0.26	0.01
6	Location of poultry farms (3)	1.2 ± 0.27	2.6 ± 0.19	1.4 ± 0.31	0.00
7	Disinfectants used in foot and vehicle bath (3)	1.4 ± 0.16	2.47 ± 0.1	1.07 ± 0.13	0.00
8	Cleaning of sheds (3)	1.9 ± 0.18	2.57 ± 0.11	0.67 ± 0.1	0.00
9	Waste water disposal (3)	1.57 ± 0.14	2.4 ± 0.12	0.83 ± 0.09	0.00
10	Ensuring chick Quality (3)	1.67 ± 0.15	2.5 ± 0.1	0.83 ± 0.12	0.00
11	Ensuring feed and water quality (3)	0.9 ± 0.25	2.2 ± 0.24	1.3 ± 0.28	0.00
12	Water sanitisation (3)	1.27 ± 0.14	2.37 ± 0.12	1.1 ± 0.1	0.00
13	Clinical signs/ diseases (3)	2 ± 0.17	2.73 ± 0.09	0.73 ± 0.12	0.00
14	Vaccination (3)	1.37 ± 0.14	2.47 ± 0.11	1.1 ± 0.08	0.00
15	Ethnoveterinary practices (3)	1.3 ± 0.15	2.3 ± 0.14	1 ± 0.07	0.00
16	Usage of antibiotics (3)	1.7 ± 0.18	2.67 ± 0.09	0.97 ± 0.16	0.00
17	Mention some antibiotics (3)	1.43 ± 0.17	2.5 ± 0.11	1.07 ± 0.13	0.00
18	Method of usage (3)	2.13 ± 0.19	2.6 ± 0.12	0.47 ± 0.09	0.00
19	AMR (3)	1.23 ± 0.18	2.57 ± 0.13	1.33 ± 0.15	0.00
20	Manure disposal (3)	1.7 ± 0.18	2.57 ± 0.11	0.87 ± 0.12	0.00
21	Dead bird disposal (3)	1.53 ± 0.19	2.53 ± 0.11	1 ± 0.13	0.00
22	Rodent control (3)	1.77 ± 0.18	2.67 ± 0.09	0.9 ± 0.16	0.00
23	Wild bird menace & control (3)	1.07 ± 0.15	2.53 ± 0.16	1.47 ± 0.12	0.00
	Overall score (69)/total marks obtained	37.07 ± 0.99	58.7 ± 0.58	21.63 ± 0.98	0.00

#### 3.2.3 Knowledge Gained on Commercial Desi Farmers

**FIGURE 3:**
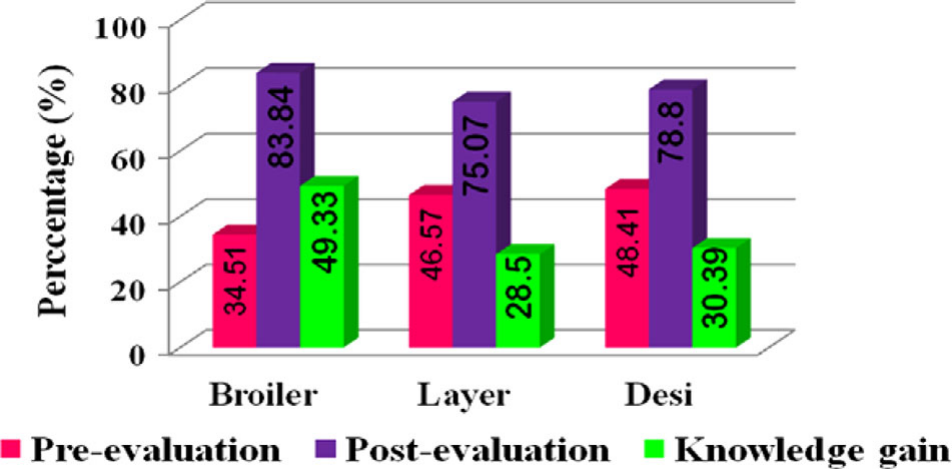
Overall training impact on pre‐existing knowledge and knowledge gain in biosecurity and management practices of poultry production system in Tamil Nadu.

Commercial desi farmers also had lower knowledge gains than commercial broiler farmers and did not reach high or moderate levels. They obtained low scores on farm fencing (1.31/3), entry of vehicles (1.09/3), disinfectants used in foot and vehicle bath (1.09/3), dead bird disposal (1.06/3), rearing practices (1.03/3), rodent control (1.03/3), ensuring feed and water quality (1.03/3), water sanitisation (1/3), and antimicrobial resistance (1/3), as presented in Table 4. However, farmers had a moderate level of score obtained in other parameters such as stocking period interval (0.94/3), vaccination (0.91/3), distance between sheds (0.84/3), location of poultry farms (0.84/3), poultry diseases (0.84/3), antibiotics (0.84/3), manure disposal (0.84/3), wastewater disposal (0.81/3), and wild bird menace and control (0.81/3). Conversely, a low score was obtained in the field of antibiotic usage (0.69/3), cleaning of sheds (0.72/3), ensuring chick quality (0.72/3), ethno veterinary practices (0.75/3), and antibiotics (0.75/3). The overall knowledge gained was more pronounced (Figure 3) among broiler farmers (49.33%), followed by desi farmers (30.39%) and layer farmers (28.5%), but there is no significant difference among these categories of farms (Table 5). Binary logistic regression analysis found that farmers' education level has a significant (*p* < 0.05) impact on disposing of dead birds, with an odds ratio of 0.39 (95% confidence interval, CI: 0.15–1.01) and knowledge gained across all farm categories (Table 6). Because educated farmers grasped their knowledge and understood the effects of poultry diseases on human welfare, they maintained a separate pit to dispose of dead birds after the training program.

**TABLE 4:** Knowledge score (mean ± SE) analysis on commercial desi farmers.

S. No.	Particulars	Mean score before the training	Mean score after the training	Difference in score	*p* value
1	Farm fencing (3)	1.03 ± 0.13	2.34 ± 0.13	1.31 ± 0.11	0.00
2	Restricted entry (3)	1.63 ± 0.13	2.72 ± 0.08	1.09 ± 0.11	0.00
3	Rearing practices (3)	1.56 ± 0.11	2.59 ± 0.10	1.03 ± 0.11	0.00
4	Distance between sheds (3)	1.78 ± 0.26	2.63 ± 0.18	0.84 ± 0.24	0.02
5	Stocking period interval between batches (3)	1.31 ± 0.27	2.25 ± 0.23	0.94 ± 0.28	0.02
6	Location of poultry farms (3)	1.50 ± 0.27	2.34 ± 0.22	0.84 ± 0.24	0.02
7	Disinfectants used in foot and vehicle bath (3)	1.34 ± 0.17	2.44 ± 0.13	1.09 ± 0.14	0.00
8	Cleaning of sheds (3)	1.97 ± 0.15	2.69 ± 0.10	0.72 ± 0.12	0.00
9	Waste water disposal (3)	1.59 ± 0.11	2.41 ± 0.12	0.81 ± 0.11	0.00
10	Ensuring chick Quality (3)	1.63 ± 0.12	2.34 ± 0.12	0.72 ± 0.08	0.00
11	Ensuring feed and water quality (3)	0.75 ± 0.23	1.78 ± 0.26	1.03 ± 0.26	0.00
12	Water sanitisation (3)	1.28 ± 0.14	2.28 ± 0.12	1 ± 0.1	0.00
13	Clinical signs/diseases (3)	2.00 ± 0.13	2.84 ± 0.07	0.84 ± 0.12	0.00
14	Vaccination (3)	1.41 ± 0.11	2.31 ± 0.11	0.91 ± 0.1	0.00
15	Ethnoveterinary practices (3)	1.38 ± 0.11	2.13 ± 0.13	0.75 ± 0.11	0.00
16	Usage of antibiotics (3)	1.59 ± 0.27	2.44 ± 0.21	0.84 ± 0.24	0.02
17	Mention some antibiotics (3)	1.78 ± 0.13	2.53 ± 0.11	0.75 ± 0.11	0.00
18	Method of usage (3)	1.47 ± 0.12	2.16 ± 0.14	0.69 ± 0.12	0.00
19	AMR (3)	0.97 ± 0.11	1.97 ± 0.13	1 ± 0.14	0.00
20	Manure disposal (3)	1.19 ± 0.08	2.03 ± 0.12	0.84 ± 0.11	0.00
21	Dead bird disposal (3)	1.34 ± 0.12	2.41 ± 0.13	1.06 ± 0.12	0.00
22	Rodent control (3)	1.41 ± 0.09	2.44 ± 0.10	1.03 ± 0.11	0.00
23	Wild bird menace & control (3)	1.50 ± 0.14	2.31 ± 0.10	0.81 ± 0.95	0.00
	Overall score (69)/total marks obtained	33.41 ± 0.77	54.38 ± 0.65	20.97 ± 0.73	0.00

**TABLE 5:** Knowledge gain (mean ± SE) assessment between different categories of poultry farmers in Tamil Nadu, India.

S. No.	Category of poultry farmers	Chi square value	df	*p* value
1	Commercial broiler vs. commercial layer	294	204	0.407^*^
2	Commercial layer vs. commercial desi	198.67	204	0.592^*^
3	Commercial desi vs. commercial broiler	233.07	204	0.079^*^

* There is no significant difference association between knowledge gain and three different category of poultry farmers (*p* > 0.05).

**TABLE 6:** Binary logistic regression used to analyse the education which influencing knowledge gain in three different categories of farms.

Univariate analysis				
Variables	Sig.	Odds ratio	95% CI	
			Lower	Upper
Farm fencing	0.851	0.92	0.38	2.18
Foot and vehicle bath	0.947	0.97	0.39	2.36
Method of rearing	0.339	0.71	0.36	1.41
Distance between sheds	0.340	0.77	0.46	1.30
Stocking period interval	0.980	0.99	0.61	1.59
Location of poultry farms	0.332	0.80	0.52	1.24
Disinfectants used in foot and vehicle bath	0.798	1.11	0.48	2.56
Cleaning of sheds	0.671	1.22	0.47	3.18
Waste water disposal	0.180	0.51	0.19	1.35
Ensuring chick quality	0.740	0.88	0.43	1.82
Ensuring feed and water quality	0.403	1.24	0.74	2.05
Water sanitisation	0.959	1.02	0.37	2.78
Poultry diseases	0.172	0.60	0.29	1.24
Vaccination	0.731	0.88	0.45	1.73
EVM practices	0.779	1.14	0.45	2.86
Antibiotics	0.404	1.26	0.72	2.20
Antibiotic treatment	0.852	0.93	0.45	1.91
Antibiotic usage	0.964	0.98	0.48	1.99
AMR	0.866	1.06	0.51	2.18
Manure disposal	0.960	1.02	0.42	2.46
Dead bird disposal	0.053*	**0.39**	**0.15**	**1.01**
Rodent control	0.165	1.91	0.76	4.76
Wild bird menace control	0.332	0.72	0.37	1.39

### 3.3 Adoption Score Analysis

Based on the knowledge interventions, the implementation of various biosecurity practices, including farm fencing, restricted entry, foot and vehicle baths, stocking density regulation, shed disinfection, water sanitisation, monitoring poultry diseases and signs, antibiotic usage as per veterinary recommendations, proper manure disposal, dead bird disposal, and rodent and wild bird control, was assessed. The level of adoption for each biosecurity practice was categorised into three levels: no adoption (1), partial adoption (2), and full adoption (3).

The adoption scores revealed that full adoption of farm fencing was highest among broiler farms at 62.96%, followed by commercial desi farms at 43.75% (Kirkpatrick level 3). Broiler and layer farms achieved full adoption rates of 55.56% and 46.67% for foot and vehicle baths with disinfectants, respectively. Broiler farms showed a 48.15% full adoption rate for restricted entry of vehicles and persons from neighbouring farms. The highest adoption rates for stocking density were observed among broiler (85.19%), and layer (73.33%) farms and chlorinated water usage reached 100% adoption in both categories of farms. Commercial desi farms lagged behind, with only 46.87% adoptions. Layer farms exhibited a higher adoption rate of (63.3%) for isolating clinically signed/disease‐infected birds in separate sheds or areas on the farm, compared to broiler farmers who had recently initiated this practice at a rate of 55.56%, albeit with no significant difference between the two categories of farms (Table 7). In addition to antibiotic usage as per veterinary recommendations, 66.67% of layers and 62.96% of broiler farms had fully adopted the prescribed practices. However, proper manure disposal was initiated by 51.85% of the broiler farmers and 43.33% of the layer farms. Meanwhile, proper dead bird disposal was undertaken by 66.67% of broilers and 53.33% of layer farmers on their own farms. Furthermore, broiler and layer farmers maintained wild bird control at similar rates, ranging from 40% to 43%, compared to commercial desi farms. The adoption rates of all biosecurity practices significantly increased (*p* > 0.05), except for rodent and pest control. The overall biosecurity practices revealed that internal biosecurity practices of stock maintenance, cleaning and disinfection of sheds, chlorination of water, disease management and vaccination, and antibiotic usage are adopted by the farmers and gradually increased at a maximum level in broiler and layer farms compared to commercial desi farms. Similarly, external biosecurity practices of farm fencing, restricted entry, foot and vehicle bath, dead bird disposal, and manure disposal were adopted at a maximum level (Kirkpatrick level 3) in the broiler and layer farms except for rodent and wild bird control.

**TABLE 7:** Adoption behaviour of biosecurity practices in three different categories of poultry farms, 3 months after the training intervention.

Biosecurity practices	Adoption	Broiler (*n* = 27)	Layer (*n* = 30)	Commercial desi (*n* = 32)	Chi value	*p* value
Farm fencing (S)	No adoption	3.70	33.33	31.25	9.764	0.045
	Partial adoption	33.33	33.33	25		
	Full adoption	62.96	33.33	43.75		
Restricted entry (S)	No adoption	14.81	16.67	46.875	12.76	0.013
	Partial adoption	37.04	26.67	31.25		
	Full adoption	48.15	56.67	21.875		
Foot and vehicle bath (S)	No adoption	0.00	20.00	37.5	16.416	0.003
	Partial adoption	44.44	33.33	43.75		
	Full adoption	55.56	46.67	18.75		
Maintenance of stock (O)	No adoption	0.00	10.00	75	52.117	0.000
	Partial adoption	14.81	16.67	15.625		
	Full adoption	85.19	73.33	9.375		
Cleaning and disinfection of sheds (O)	No adoption	0.00	10.00	34.375	18.811	0.001
	Partial adoption	44.44	23.33	37.5		
	Full adoption	55.56	66.67	28.125		
Use of disinfectants in water (O)	No adoption	0.00	0.00	28.125	37.431	0.001
	Partial adoption	0.00	0.00	25		
	Full adoption	100.00	100.00	46.875		
Poultry diseases and vaccination (O)	No adoption	3.70	6.67	62.5	40.083	0.001
	Partial adoption	55.56	30.00	21.875		
	Full adoption	40.74	63.33	15.625		
Antibiotic usage (O)	No adoption	7.41	13.33	56.25	29.166	0.001
	Partial adoption	29.63	20.00	31.25		
	Full adoption	62.96	66.67	12.5		
Manure disposal (S)	No adoption	3.70	16.67	65.625	31.234	0.001
	Partial adoption	44.44	40.00	21.875		
	Full adoption	51.85	43.33	12.5		
Dead bird disposal (O)	No adoption	0.00	16.67	62.5	32.851	0.001
	Partial adoption	33.33	30.00	21.875		
	Full adoption	66.67	53.33	15.625		
Rodent control (S)	No adoption	22.22	20.00	43.75	5.768	0.217
	Partial adoption	44.44	43.33	37.5		
	Full adoption	33.33	36.67	18.75		
Wild bird control (S)	No adoption	14.81	30.00	68.75	21.25	0.000
	Partial adoption	44.44	26.67	9.375		
	Full adoption	40.74	43.33	21.875		

O, operational biosecurity practices; S, structural biosecurity practices.

## 4 Discussion

India has diverse poultry production systems, and this study provided a better understanding of farmers' perceptions of biosecurity and the challenges they may face when implementing biosecurity measures. Implementing biosecurity is crucial for successful poultry production, especially in maintaining a disease‐free environment and preventing outbreaks such as highly pathogenic avian influenza followed by non‐typhoidal salmonella and campylobacter species. Delpont et al. (2023) and Thames and Sukumaran (2020) also reported a similar statement. However, farmers must understand its key principles to embrace biosecurity practices effectively. This knowledge alters farmers' perceptions of disease risks and enhances their awareness of the critical role of biosecurity measures. Several studies have recommended training poultry farmers in strict biosecurity protocols to mitigate HPAI in India (Mahadevan et al. 2024). In Tamil Nadu, this is the first report about farmers' knowledge of and implementation of biosecurity practices performed on broiler, layer, and commercial desi farms. This study provides baseline information on the demographic and socioeconomic profiles of the farmers owning chicken farms, the impact of training intervention on poultry farming management and biosecurity practices (both theoretical and practical sessions), and the implementation of biosecurity practices among chicken farms in the three major poultry producing regions of Tamil Nadu.

The present study found that males dominated females in terms of participation in the training program, which showed more significant variation within each category of Indian poultry farming. Male farmers were mainly involved in modern poultry farming, like layers and broilers, except in commercial desi farms. Women in rural and urban regions of Tamil Nadu recently operated commercial desi farms. Okoh et al. (2010) reported that men participate more in poultry management activities such as vaccination, drug delivery, debeaking, and supplying chicks, while women are only involved in daily routines like cleaning cages, providing potable water, sorting eggs, etc. In India, modern poultry farming is more profitable than village chicken rearing for women. Men prefer to work in contemporary poultry farming rather than traditional chicken farming. Ajewole and Akinwumi (2014) and Kouam (2019) found that most small‐scale broiler farms were operated by men. This statement is also supported by Adam et al. (2014) and Meher et al. (2020).

This study also revealed that 89.9% of the farmers belonged to the 21–60 years of age group. This suggests that younger individuals mainly work in poultry production and have a secondary educational background. It is a common consensus that educated farmers will have more information regarding poultry production and disease management (Adam et al., 2014; Hamid et al. 2018). Moreover, most farmers obtained revenue from the combined activities of poultry farming and agriculture, followed by poultry farming alone. This statement reveals that most farmers own agricultural land (Islam et al. 2015). However, 56.2% of farmers had 5 to 20 years of experience in the poultry sector, and they learned more practices with farming and management practices. This could be because more experienced farmers can identify symptoms in the earlier days and treated than the less experienced farmers. Choudhuri et al. (2010) observed that trained and experienced farmers are well‐established in poultry farming because of their knowledge gained. Our findings also corroborate this statement. The majority of poultry farms reared smallholder farms (93.3%) with less than 25,000 birds. However, only 6.7% of farmers have reared larger farms (25,000–100,000 birds), and they follow strict biosecurity measures compared to smallholder farms, which indicates that they operate small and medium‐sized farms. Hamid et al. (2016) and Das et al. (2024) observed that small and medium‐scale farms (81%) continue to dominate commercial broiler production in Bangladesh.

The knowledge mean score results showed that most poultry farmers must know about antibiotic usage, antimicrobial resistance, manure disposal, and wild bird management in broiler farms. They had scored very low in the pre‐training evaluation. However, after the training program, knowledge gained was high in these topics. Based on the knowledge level at the time of pre‐evaluation, the desi chicken farmers had some basic knowledge regarding all the management practices compared to broiler farmers. This could be due to the training programs offered by social organisations and agricultural extension departments for commercial desi chicken farmers and also to enhance women entrepreneurs. There needed to be a significant gap in knowledge level on feed and water quality, antimicrobial resistance, and farm fencing in each category of poultry farms. Farm fencing is necessary for commercial desi chicken production (Miao et al. 2005), which practices a free‐ranging system. Birds move from the sheds to nearby farms and come in contact with wild rodents, feral birds, and insects. In addition, feed and water quality are of utmost importance to the poultry industry (Zampiga et al. 2021). This is essential for deciding the birds’ performance, nutrition, antibiotics, and disease‐free environment.

For this reason, the training team focused on this topic, which led to good knowledge gain. Overall, the results revealed that the farmers had gained more knowledge on farm fencing, antimicrobial resistance, and ensuring feed and water quality. Desi farmers are independent decision‐makers on their own farms and get information from the agricultural extension departments. By contrast, most broiler farms are integrated farms, and they follow regulations regarding vaccines, antibiotics, and feed from farm supervisors who also monitor the farms. According to the present investigation, farmers had existing knowledge about biosecurity practices like rearing, the distance between sheds, foot and vehicle baths, water sanitisation, and ensuring chick quality before the training program. Because these are all routine practices, they have carried out on their farms with some experience. This led to lower knowledge gain, indicating a meagre difference in knowledge between pre‐ and post‐training interventions in all three categories of farmers. Despite this, due to awareness, smallholder farmers must follow strict biosecurity practices (Mudenda et al. 2022). Adam et al. (2014) also reported that poor awareness could imply low hygienic practices and poor understanding of farm disease dynamics.

In this study, poultry farmers were trained and given field demonstrations to increase awareness and implement biosecurity practices on their farms within 3 months. This was identified by the adoption behaviour of their corresponding farms. A similar finding was already reported by Bedekelabou et al. (2022). This awareness level reflects educational status, with 87.7% of respondents having secondary and tertiary levels of education. By contrast, Ferdous et al. (2019) reported that most of the farmers had master‐level education and frequently practiced personal hygiene practices on their farms.

The adoption levels of biosecurity practices were analysed in the three categories of farms. Of these, broiler and layer farms have adopted a maximum percentage of all biosecurity practices, including internal and external control, when compared to commercial desi farms. The low percentages of positive responses regarding various biosecurity measures highlight significant risk factors. These include vehicles entering farm premises without proper washing and disinfection, farm workers residing off‐site, lack of segregation of chickens by age, and utilisation of second‐hand equipment. The risks associated with disease outbreaks include receiving visitors on farms, inadequate rodent control and wild bird management, absence of proper storage facilities, and failure to conduct on‐farm necropsies. These risks not only facilitate the spread of contagious poultry diseases by human contact but also pose public health concerns regarding zoonotic diseases such as avian influenza, as noted by Hassan et al. (2016).

The structural biosecurity measures in poultry farms refer to the physical and operational strategies implemented to prevent pathogen introduction and spread within and between farms. Implementing these measures in India has become increasingly important due to recurring outbreaks of avian influenza (bird flu), Newcastle disease, and other zoonotic diseases (Bleich et al. 2009). To avoid these circumstances, implementing structural biosecurity is essential for disease‐free flocks. Structural biosecurity is one of the significant challenges in India is the high initial cost of implementing structural biosecurity measures, particularly for smallholder farmers. Because many small farms may lack the financial resources to invest in advanced structural modifications like biosecure fencing, proper ventilation systems, and automated monitoring (Negro‐Calduch et al. 2013).

The prevalence of diseases was the primary constraint in the investigated smallholder farms, and they faced a lot of consequences of inadequate biosecurity implementation. However, the low level of biosecurity score for traffic onto the shed is caused by many people being able to enter the shed and rodents. This was evident from some consumers bought eggs directly in the shed. Human activities were known to be the main route for spreading diseases (Bleich et al. 2009; Yoo et al. 2022). The other evidence was rodents entering the shed. Backhans and Fellstrom (2012) argued that farm rodents are dangerous to introducing new infections into the livestock, so rodent control should be considered an important measure to provide good bio‐security. When comparing these findings with research conducted by Susilowati et al. (2010), it was observed that the majority of layer smallholders in West Java (49%) had adopted high levels of biosecurity measures, indicating a better implementation of biosecurity practices in their farms. East (2007) also stated that most chicken farms in commercial layers in Australia had adopted high levels of biosecurity and hygiene practices.

## 5 Conclusions

Based on the results, it can be concluded that the level of biosecurity adoption on poultry farms in Tamil Nadu was categorised as ‘partial cum full adoption’. Veterinarians or social workers in agricultural extension departments should intensify efforts to provide information about good management practices to the significant poultry production regions. Participatory training has significantly improved knowledge on the poultry management and biosecurity practices. However, limited adoption of biosecurity practices and changes in the attitude of some smallholder farmers towards the implementation were observed. Farm fencing, cleaning of sheds, water sanitisation, ensuring chick quality, antimicrobial resistance, monitoring poultry diseases, disposal of dead birds, manure management, and controlling wild bird menace are essential factors influencing farmers' knowledge and adoption behaviours regarding biosecurity. This study provides empirical evidence of the impact of training intervention on biosecurity practices for disease prevention and control.

## Author Contributions

**Funding acquisition**: Fiona Tomley. **Conceptualisation**: Ayona Silva Fletcher; Papaiyan Kumaravel. **Conceived and design**: Ayona Silva Fletcher, Vasudevan Gowthaman, Thippichettipalayam Ramasamy Gopala Krishna Murthy. **Investigation**: Vasudevan Gowthaman, Samuel Masilamoni Ronald, Papaiyan Kumaravel. **Writing – original draft**: Alagarsamy Alagesan, Ayona Silva Fletcher, Vasudevan Gowthaman. **Data curation**: Alagarsamy Alagesan, Ganesh Janarthanan, Arumugam Balakrishnan, Paramasivam Vigneshwaran.

## Ethics Statement

**1. Institutional Review Board Statement**

The current study directly involves studies with people using questionnaire‐based data collection conducted after gaining informed consent from respondents and farm owners.

**2. Informed Consent Statement**

The study was reviewed at Institution level by domain experts from the Veterinary and Animal Sciences, including the research approval committee of Tamil Nadu Veterinary and Animal Sciences University (TANUVAS). The project did not use animals but involved poultry farmers on a volunteer basis, with their informed agreement obtained after being briefed about the study's aims and the data to be gathered. Respondents were informed that all farm information is kept confidential, that no names or subject‐identifiable information would be made public, and that the information would only be used for research and publication purposes.

## Conflicts of Interest

The authors declare that they have no competing interests

### Peer Review

The peer review history for this article is available at https://www.webofscience.com/api/gateway/wos/peer‐review/10.1002/vms3.70385.

## Data Availability

For this investigation, we tried to collect primary data from commercial poultry farms in three zones. As a result, the primary data is now available to the researchers, and they can access it at any time.
